# Renal Cell Carcinoma as a Metabolic Disease: An Update on Main Pathways, Potential Biomarkers, and Therapeutic Targets

**DOI:** 10.3390/ijms232214360

**Published:** 2022-11-18

**Authors:** Nicola Antonio di Meo, Francesco Lasorsa, Monica Rutigliano, Davide Loizzo, Matteo Ferro, Alessandro Stella, Cinzia Bizzoca, Leonardo Vincenti, Savio Domenico Pandolfo, Riccardo Autorino, Felice Crocetto, Emanuele Montanari, Marco Spilotros, Michele Battaglia, Pasquale Ditonno, Giuseppe Lucarelli

**Affiliations:** 1 Urology, Andrology and Kidney Transplantation Unit, Department of Emergency and Organ Transplantation, University of Bari “Aldo Moro”, 70124 Bari, Italy; 2 Division of Urology, European Institute of Oncology, IRCCS, 20141 Milan, Italy; 3 Laboratory of Human Genetics, Department of Biomedical Sciences and Human Oncology, University of Bari “Aldo Moro”, 70124 Bari, Italy; 4 Division of General Surgery, Polyclinic Hospital, 70124 Bari, Italy; 5 Division of Urology, VCU Health, Richmond, VA 23298, USA; 6 Department of Neurosciences, Reproductive Sciences and Odontostomatology, University of Naples “Federico II”, 80131 Naples, Italy; 7 Department of Clinical Sciences and Community Health, University of Milan, 20122 Milan, Italy

**Keywords:** renal cell carcinoma, metabolomics, metabolism, Warburg effect, biomarker

## Abstract

Clear cell renal cell carcinoma (ccRCC) is the most frequent histological kidney cancer subtype. Over the last decade, significant progress has been made in identifying the genetic and metabolic alterations driving ccRCC development. In particular, an integrated approach using transcriptomics, metabolomics, and lipidomics has led to a better understanding of ccRCC as a metabolic disease. The metabolic profiling of this cancer could help define and predict its behavior in terms of aggressiveness, prognosis, and therapeutic responsiveness, and would be an innovative strategy for choosing the optimal therapy for a specific patient. This review article describes the current state-of-the-art in research on ccRCC metabolic pathways and potential therapeutic applications. In addition, the clinical implication of pharmacometabolomic intervention is analyzed, which represents a new field for novel stage-related and patient-tailored strategies according to the specific susceptibility to new classes of drugs.

## 1. Introduction

Renal cell carcinoma (RCC) is among the top 10 malignancies affecting adults globally. Statistical reports estimated that in 2022, 79,000 new cases will be diagnosed, and 13,920 patients will die of kidney cancer in the USA [1].

Clear cell renal cell carcinoma (ccRCC) is the most frequent histological kidney cancer subtype, accounting for more than 75% of all RCC cases [2]. RCC derives its “clear cell” name from the histological appearance of cells, which consists of thin-walled cells filled with abundant lipids and glycogen. In the early stages, this disease is frequently asymptomatic, and incidentally, diagnosed by imaging, having a good prognosis. Conversely, ccRCC has a high mortality rate in the advanced stage due to poor responses to radiotherapy and chemotherapy [3,4,5]. Over the last decade, significant progress has been made in identifying the genetic alterations driving ccRCC development [6,7]. High-throughput sequencing databases such as the Catalogue of Somatic Mutations in Cancer (COSMIC) and The Cancer Genome Atlas (TCGA) project provided a clear overview of the genes frequently mutated in ccRCC [8,9]. In particular, a total of 13 RCC predisposing genes (VHL, MET, BAP1, TFE3, TFEB, FLCN, MITF, FH, SDHB, SDHC, SDHD, TSC1, and TSC2) have been identified through studies of hereditary kidney cancer syndromes. Other genes significantly mutated in sporadic ccRCC, in addition to VHL, include PBRM1, SETD2, and KDM5C [10,11,12,13,14,15,16,17,18,19,20,21,22,23]. Interestingly, these three genes encode for proteins with an important role in the maintenance and remodeling of chromatin states. The initiation and development of ccRCC are, in fact, closely related to genomic alterations by epigenetic modification, such as DNA methylation, noncoding RNAs, and post-translational histone modifications.

Furthermore, it has been made clear that epigenetic changes affect the metabolic shift in ccRCC and that, in turn, metabolic intermediates may have an impact on how the genome is expressed during ccRCC progression. A recent study showed that RCC significantly overexpressed SETD8, which was strongly associated with lipid storage, advanced tumor grade and stage, and poor prognosis [24]. Conversely, the increased level of the oncometabolite 2-hydroxyglutarate has been associated with epigenetic modifications which promote a neoplastic phenotype [25].

Many authors analyzed genetic and pathological features and revealed that aberrant metabolism is recurrent in ccRCC cells. These findings led to widespread metabolic profiling studies, intending to define a relationship with cancer behavior in terms of aggressiveness, prognosis, and therapeutic responsiveness [26,27,28,29,30,31].

Moreover, recent studies have also revealed a crucial role of noncoding RNAs (ncRNAs) in the regulation of different processes of neoplastic cells. Specifically, long noncoding RNAs (lncRNAs), which are large RNA transcripts more than 200 nucleotides in length, are aberrantly expressed in many human cancers, control cellular energy metabolism, and have integrative functions in cancer cells’ pathogenesis and development. In recent years, the integration of their role in ccRCC metabolism revealed their potential in biomarker detection and therapeutic-targeting research. However, further research is still required to fully understand the numerous unanswered questions on the biological functions of lncRNAs [32]. In a previous article [33], we reviewed the different ccRCC-altered pathways based on an in-depth analysis of the available data on the ccRCC metabolome.

In this review article, we provide an update on the current state-of-the-art in ccRCC metabolome research, integrating information acquired from to lncRNAs studies and evaluating the possibility of uncovering novel therapeutic targets.

## 2. Metabolic Pathway Understanding: Where Do We Stand?

### 2.1. Lactate Metabolism

The Warburg effect was been one of the earliest sources of evidence of metabolic reprogramming for ccRCC. Even in the presence of abundant oxygen availability, most cancer cells produce energy predominantly by glycolysis, whereas most normal cells produce energy by mitochondrial oxidative phosphorylation. This tumor-specific Warburg effect provides the energy and biosynthetic substrates needed to promote tumor progression [34].

The Warburg effect involves the modification of metabolic enzymes, such as hexokinase 2 (HK2), pyruvate kinase type M2 (PKM2), glucose transporter 1 (GLUT1), lactate dehydrogenase (LDH), and lactate transporters (monocarboxylate transporters (MCTs)) [35,36,37], as well as the activation of numerous transcription factors, including c-Myc, NF- κB, and hypoxia-inducible factor 1α (HIF-1α) [34,36,37,38,39,40]. In ccRCC, VHL/HIF activity significantly alters cell glucose input and utilization [41,42,43]. The inactivation of VHL in ccRCC leads to the aberrant accumulation of the transcription factors hypoxia-inducible factor (HIF)-1α and HIF-2α, despite normoxia with the resultant upregulation of pathways involved in glycolysis, fatty acid (FA), and glycogen synthesis [41,42,43] promoting a lactate-rich environment [34,44,45] (Table 1).

Lactate, once considered a byproduct of anaerobic glycolysis, plays a crucial role in tumor formation, maintenance, and treatment response [45,46,47,48,49,50,51,52,53]. Several investigations have linked lactate generation to tumor growth and resistance to therapy, angiogenesis, metastasis, immunological evasion, and radio-resistance [54,55]. There is also growing evidence linking the overexpression of lactate dehydrogenase A (LDHA) to the growth of tumor cells.

Zhao et al. [56] determined through in vitro and in vivo investigations that LDHA improves RCC tissues and accelerates tumor migration and invasion via the epithelial–mesenchymal transition (EMT). In addition, a high level of LDHA may act as a predictor of poor outcomes in individuals with RCC [57].

Recently, Sun et al. [58] investigated the global influence of lactate-related genes (LRGs) on prognostic significance, tumor microenvironment characteristics, and therapeutic response. They analyzed data from The Cancer Genome Atlas (TCGA), E-MTAB-1980, and GSE22541 cohorts and identified 17 differentially expressed LRGs, 3 of which were determined to construct a lactate-related prognostic signature (LRPS) using LASSO and Cox regression analyses. This signature could classify patients with RCC into low- and high-risk groups, and showed a robust survival prediction efficiency.

The signature included three genes (HADH, FBP1, and TYMP) with a fundamental role in the development of different malignancies.

The short-chain L-3-hydroxy acyl-CoA dehydrogenase (HADH) gene, which encodes the HADH enzyme, is involved in the third step of FA β-oxidation [59]. Its overexpression seems to be related to poor clinical outcomes, cell migration, and invasion in acute myeloid leukemia, colon cancer [60,61], and gastric cancer [62]. A reduced expression of HADH was associated with immune infiltration and a poor prognosis in RCC patients [63].

Fructose-1,6-bisphosphatase 1 (FBP1) is a gluconeogenic rate-limiting enzyme that could regulate the uptake of glucose and the secretion of lactate by alleviating the level of glycolysis and NADPH in RCC cells under the influence of HIFs.

Thymidine phosphorylase (TYMP), a platelet-derived endothelial cell growth factor, is overexpressed in tumor cells and surrounding extra matrix cells. It catalyzes the reversible phosphorolysis of thymidine [64] and is upregulated in multiple solid tumors [65]. This protein was associated with the expression of a number of immunocyte markers and may have a function in controlling the immunological response, indicating its potential relevance as an immunotherapy target [64,66].

Furthermore, LRPS-based risk groups showed different immune components with significant differences in prognosis and immune cell infiltration. Moreover, the tumor metabolic symbiosis hypothesis [67], which asserts that lactate may be employed as an energy source in the tumor microenvironment [34], has been put forth. Miranda-Gonçalves et al. [68] showed that increased extracellular lactate, exported by MCTs, decreases sirtuin-1 (SIRT1) expression and activity. SIRT1 is an NAD+-dependent class III histone deacetylase involved in RCC epigenetic mechanisms. In their findings, decreased SIRT1 activity resulted in increasing global H3 and H3K9 acetylation.

Due to SIRT1 inhibition, lactate showed a reversible influence on histone deacetylation levels and caused histone hyperacetylation in vitro and in vivo. Previous studies showed that histone acetylation was involved in the transcriptional regulation of EMT-related genes and associated pathways [69,70,71]; furthermore, other results suggest that SIRT1 suppresses the EMT and metastasis process by deacetylating SMAD4 [72,73].

SMAD proteins are a family of intracellular mediators that control TGF-superfamily signaling, and SMAD4 is one of its members. It functions as a convergent node in the SMAD pathways that are located downstream of the TGF-β RI and TGF-β RII receptors. In order to bind particular DNA sequence motifs and modify the expression of target genes, it can interact with all R-SMAD proteins [74,75].

According to Lichner Z et al. [76], SMAD4 plays a critical role in enhancing EMT activation and stem cell-like characteristics of RCC cell lines.

Interestingly, in Miranda-Gonçalves’ study, treatment with nicotinamide (NAM), a noncompetitive SIRT1 inhibitor, paralleled lactate effects and promoted cell aggressiveness. On the other hand, alpha-cyano-4-hydroxycinnamate (CHC), a lactate transporter inhibitor, reduced cancer cell migration and attenuated the malignant phenotype. The authors explained how lactate exposure or NAM treatment decreased SIRT1 and enhanced N-cadherin and vimentin expression in RCC cell lines.

**Figure 1:**
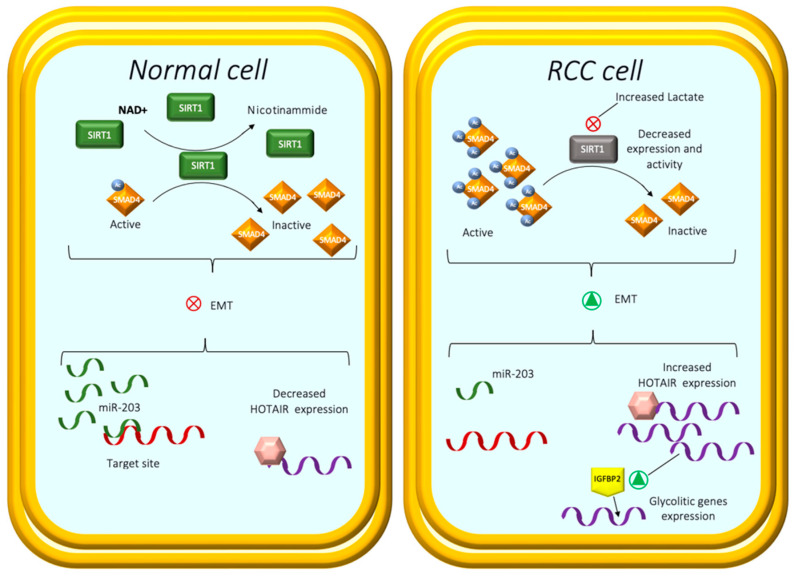
In normal cells, SIRT1 suppresses EMT and metastasis process by deacetylating SMAD4. In RCC, decreased SIRT1 activity and subsequent increased β-catenin protein levels promote an EMT-invasive phenotype. The expression levels of miR-203 in RCC cancer cells are lower than in normal cell lines. miR-203 binds toa target site and attenuates HOTAIR expression. HOTAIR enhances RCC cell migration by regulating the IGFBP2 expression. The HOTAIR–IGFBP2 axis induces glycolytic gene expression, correlating with increased proliferative and migratory capacity.

Moreover, they observed high SMAD4 acetylation levels after lactate and NAM treatment due to decreased SIRT1 activity and increased β-catenin protein levels, promoting an EMT-invasive phenotype (Figure 1).

In addition, it was demonstrated that the exposure of normal kidney cell lines to lactate and a tumor cell-conditioned medium resulted in cellular changes identical to those identified in tumor cells, and hence, contributed to the pseudo-transformation of nearby normal cells and the aggressiveness of RCC. Focusing on EMT, further different approaches have been conducted to obtain new details about this shift. Dasgupta et al. [77] investigated the role of miR-203, a regulatory microRNA, in the regulation of lncRNA Hox antisense intergenic RNA (HOTAIR). According to these findings, the expression levels of miR-203 in RCC cell lines and patient tumor samples were lower than in normal cell lines and normal tissue, respectively. In contrast, HOTAIR had higher expression levels in RCC cell lines and clinical samples than their matched normal tissues or normal cell lines. They described how miR-203 binds to a target site and attenuates HOTAIR expression in renal cancer cells. They found that miR-203 overexpression in RCC cells increased the epithelial markers E-cadherin and claudin, with a concomitant decrease in the mesenchymal marker vimentin at both the mRNA and protein levels. Thus, this report envisages that miR-203 and HOTAIR may be helpful in RCC therapeutics.

Katayama H et al. [78] investigated the role of HOTAIR and demonstrated that HOTAIR enhances RCC cell migration by regulating the insulin growth factor-binding protein 2 (IGFBP2) expression. The HOTAIR–IGFBP2 axis induces glycolytic gene expression and maintains a high flow of glycolysis in ccRCC cells, which correlates with their proliferative and migratory capacity and may represent a specific therapeutic target for ccRCC.

### 2.2. The Leading Role of Transporters

The altered expression of glucose transporters is one of the major phenotypic characteristics of RCC. Renal cells express multiple members from the GLUT family of passive glucose transporters., e.g., GLUT1 and GLUT2 [79].

The increased GLUT1 expression in RCC cells is associated with the reduction in infiltrating CD8+ T cells, indicating a role of GLUT1 in the immune-escape machinery of the renal cancer cells [80]. Furthermore, this lower infiltration of CD8+ T cells might be caused by increased lactate formation due to enhanced lactic acid fermentation in the RCC cells, as lactate hinders T-cell activity [81].

The premise for diagnosis and staging by positron emission tomography employing (18F) fluorodeoxyglucose (18F-FDG PET) is the acceleration of glucose buildup via GLUTs in cancer cells [82,83]. However, 18F-FDG PET is unable to identify certain malignancies, including RCC, and its use for diagnosis and staging is not generally advised [83,84]. Recent investigations have suggested that sodium-glucose transporters (SGLTs), an additional class of glucose transporters not detectable by 18F-FDG PET, may contribute to glucose consumption in these malignancies. SGLT-1 and SGLT-2 are expressed in multiple cancer types (including RCC) and might even be involved in glucose transport in tumor tissue [85,86,87,88,89,90,91].

Recently, Kobayashi et al. [83] examined SGLT expression via immunohistochemistry in RCC tissues and suggested that elevated SGLT-2 expressions could be related to unfavorable outcomes.

An immunohistochemical analysis of 68 RCC tissue specimens showed that increased SGLT2 expression was significantly associated with shorter overall survival (OS) (*p* < 0.01), regardless of metastatic status.

Genes encoding lactate transporters, e.g., MCT1 and MCT4, are often upregulated in aggressive renal tumors [92,93].

Kim et al. provided a simultaneous analysis of MCT1 and MCT4 in ccRCC and demonstrated that overexpression predicted progression-free survival [92]. A recent study confirmed these results for MCT1 and, for the first time, directly correlated MCT1 and GLUT1 with the tumor grade, consistent with that reported previously for cervix carcinoma [94]. This central role of MCT1 and GLUT1 also concerns prognosis, with the mRNA level of both correlated with OS.

In 2021, using a public database, Yoo et al. [95] evaluated the link between the expression level of MCT members in renal cell cancers and the survival rate of patients.

MCT9 was found to be widely and selectively expressed in human kidney cells, but its expression was drastically reduced in renal malignancies. Moreover, its overexpression inhibited the growth of RCC cells. MCT9 was identified as a transporter for carnitine rather than urate [96]. Since the majority of free carnitine is totally reabsorbed by the kidney, it is possible that MCT9, the most common MCT member, contributes to carnitine transport in normal renal cells. Hence, a significant decrease in MCT9 expression in renal tumors may completely abrogate its function, which may be associated with dysregulated energy balance and muscle loss during tumor progression.

### 2.3. The Oncogenic Role of G6PD

G6PD dysregulation has been reported in RCC and various types of human cancers, and elevated levels of G6PD in association with higher levels of PPP-derived metabolites suggests a prominent role of this pathway in RCC-associated metabolic alterations [46,97,98].

Many authors showed that a high expression of G6PD in ccRCC predicted poor outcomes for ccRCC patients, identifying the oncogenic role of G6PD in RCC.

Through in vitro and in vivo analysis, Zhang et al. [99] investigated the impact of ROS accumulation on the NF-kB signaling pathway, pSTAT3, and G6PD in ccRCC.

They triggered or inhibited ROS production in RCC cell lines and observed that the G6PD mRNA expression level was increased or decreased, respectively. These results were consistent with a previous study using different ccRCC cell lines, which reported that ROS, cytokines, and other stresses could stimulate the continuous activation of pSTAT3, NF-kB, and MAPK signals [99,100,101,102,103,104] and promote the occurrence and development of RCC [100,105,106].

It was shown that pSTAT3 and NF-kB might regulate a range of tumorigenesis-related genes either synergistically or independently [107]. Overactivation of NF-kB signaling has been shown to promote RCC oncogenic transformation [108]. High pSTAT3 expression is anticipated to be an independent prognostic molecule for individuals with ccRCC [109,110]. In numerous physiological and pathological processes, such as B-cell activity [111], cancer cell nutrition [107,112], and mitochondrial fusion [113], NF-kB and STAT3 signaling pathways were found to interact. In addition, G6PD might accelerate the growth of ccRCC by enhancing ROS generation and pSTAT3 signaling activity, and pSTAT3 demonstrated a positive feedback control of G6PD transcription [100].

Therefore, Zhang et al. demonstrated that significantly reduced or increased activities of the NF-kB signaling pathway were found in ccRCC cells following treatment with an ROS scavenger or stimulator, respectively, which was consistent with the changes in pSTAT3 signaling activity and G6PD expression. According to the results presented above, NF-kB signaling and pSTAT3 were transcriptional regulators that may have a cooperative effect on G6PD overexpression in ccRCC. Following a thorough analysis, it was determined that ROS caused an overactivation of NF-kB and pSTAT3 signaling. These two signaling pathways activated each other and formed a transcriptional complex including pSTAT3 and p65, instead of p50. The p65/pSTAT3 complex occupied the pSTAT3-binding site on the G6PD transcription promoter and synergistically facilitated G6PD overexpression, contributing to ccRCC proliferation.

Different mechanisms were discovered by the same authors who recently described, through their in vitro experiments on RCC cell lines, the regulatory effect of SIRT2 on G6PD in RCC [114].

Firstly, they observed, according to previous studies, that SIRT2 was highly expressed and played an oncogenic role in ccRCC.

Then, through the coimmunoprecipitation (Co-IP) assay, they observed that SIRT2 enhanced glucose 6-phosphate dehydrogenase (G6PD) activity by deacetylation. Furthermore, western blot assays with glutaraldehyde crosslinking showed that G6PD active dimer formation was significantly increased with SIRT2 overexpression, whereas G6PD monomer formation showed opposite results.

In addition, they observed that SIRT2 overexpression favored G6PD protein stability in ccRCC by reducing G6PD ubiquitination and enhancing small ubiquitin-related modifier 1 (SUMO1). In their experiments, G6PD activity and G6PD dimer formation decreased significantly after treatment with SUMOylation inhibitor 2-D08.

In recent studies, Zhang et al. [114,115,116] also illustrated the association between G6PD activity and RCC progression, proliferation, and the migration rate, focusing on MMP2 and MMP9 activity and expression.

Due to their ability to degrade the essential elements of basement membranes, MMP2 and MMP9, also known as gelatinase A and gelatinase B, are thought to be the significant MMPs involved in the invasion and metastasis of several cancers. They discovered a positive correlation between G6PD and MMP2 by using a reverse transcription-quantitative PCR, Western blotting, enzyme activity assay, and immunohistochemistry analysis in cell and murine models, and in human specimens [116].

However, in 2021 [117], they observed no significant difference in MMP2 expression in the analysis of large numbers of ccRCC clinical samples and normal kidney tissue, and no RCC prognosis association [118], indicating that G6PD-mediated ccRCC progression may be dependent on other more critical underlying mechanisms. Subsequently, they studied the correlation between the expression patterns of G6PD, Cyclin E1, and MMP9 in ccRCC to identify relationships between clinicopathological characteristics of ccRCC and the genes of interest in the prognosis of ccRCC patients.

G6PD, Cyclin E1, and MMP9 were found to be overexpressed in ccRCC and to have a positive correlation, according to a variety of in vitro cytological function studies and xenografted murine models. Additionally, they were associated with patients with ccRCC having a bad prognosis. Additionally, G6PD altered the dynamics of the cell cycle, aided cell proliferation and migration in vitro, and improved the formation of ccRCC in vivo, most likely by promoting Cyclin E1 and MMP9 expression.

These findings revealed the feasibility of G6PD, Cyclin E1, and MMP9 being novel biomarkers and paved the way for developing novel therapeutics for ccRCC.

ROS accumulation in RCC activates the NF-kB signaling pathway, pSTAT3, and G6PD, which regulate tumorigenesis-related genes and promote RCC oncogenic transformation. These two signaling pathways activated each other and formed a transcriptional complex that includes pSTAT3 and p65. The p65/pSTAT3 complex occupies the pSTAT3-binding site on the G6PD transcription promoter and synergistically facilitates G6PD overexpression.

**Figure 2:**
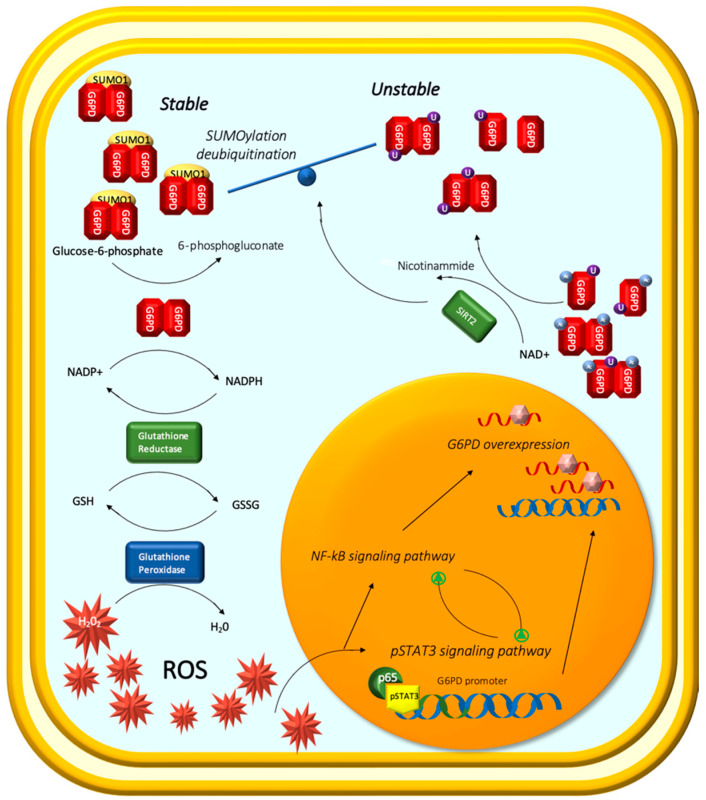
High levels of G6PD suggest that this pathway plays a major role in metabolic changes caused by RCC.

SIRT2 is highly expressed in RCC and enhances G6PD activity and stability balance through deacetylation, dimer formation, and SUMOylation (Figure 2).

### 2.4. The Metabolic Background of Lipid Droplets

Solid tumor cells require extracellular FA as a nutrient source, especially under metabolic stress conditions [119], and the dysregulation of lipid metabolism is among the most prominent changes in ccRCC.

A hallmark of ccRCC is the accumulation of cholesterol, cholesterol esters, and other lipids collected in intracellular lipid droplets (LDs) [49,120]. These dynamic organelles are responsible for lipid uptake and storage, homeostasis support, energy production, and membrane biogenesis during rapid tumor cell growth and transformation [121,122,123,124]. Furthermore, a decrease in the expression of specific FA β-oxidation enzymes has also been correlated with an increase in tumor stage, size, and grade, with a concomitant decrease in survival [125,126].

Recent studies tried to explain the metabolic and genetic background of lipid storage and droplet formation, known as a grade-dependent phenomenon [127].

When cells are under stress, LDs play a crucial role in maintaining energy and redox balance, controlling autophagy, preserving ER homeostasis, and defending cells against lipotoxicity [121]. By buffering cellular lipid saturation through the interchange of TG-resident unsaturated FA in ccRCC, LDs reduce the buildup of toxic, saturated lipids under hypoxia conditions [122].

Recently, it has been shown that two LD-associated proteins, lipid droplet protein perilipin 2 (PLIN2) and hypoxia-inducible lipid droplet-associated (HILPDA), are overexpressed in ccRCC, where they regulate lipid storage and enrich lipids that contain polyunsaturated fatty-acyl side chains [128,129]. The phospholipid-binding protein annexin A3 (AnxA3) has been described as a negative regulator of adipocyte differentiation and is downregulated in RCC. It reveals a differential expression pattern for two isoforms of 36 and 33 kDa. Bombelli et al. [129] investigated the involvement of AnxA3 isoforms in the lipid storage modulation of ccRCC cells. They found that the increased accumulation of lipids in ccRCC cells correlated with a decrease in the 36/33 isoform ratio, and 36-kDa AnxA3 silencing in ccRCC cells increased lipid storage induced by an adipogenic medium. The subcellular distribution of AnxA3 in the cellular endocytic compartment suggested that it may negatively modulate lipid storage in ccRCC cells by interfering with the caveolin-1-dependent vesicular trafficking involved in lipid uptake and accumulation in ccRCC.

Cholesterol plays an essential role in controlling membrane fluidity and assembly and the function of lipid rafts, which contain multiple signaling cascades such as RAS, AKT, and SRC that are involved in cancer development [130,131].

Cholesterol arrives in cells through direct uptake from the diet and can be acquired through the receptor-mediated uptake of plasma lipoproteins. The low-density lipoprotein receptor (LDL-R), very low-density lipoprotein receptor (VLDL-R), and scavenger receptor B1 (SR-B1) are the major receptors involved in exogenous cholesterol uptake [132,133]. Once a lipoprotein binds to its receptor, it forms an endosome within the membrane, which translocates into cells. Following internalization and transport into the lysosome, the cholesterol ester is hydrolyzed by lysosomal acid lipase (LAL) to release the free cholesterol [134].

In ccRCC tissues, VLDL-R and LAL are upregulated and associated with lower patient survival [50,135,136].

Cells can also synthesize de novo cholesterol through the mevalonate pathway. This pathway starts with the condensation of acetyl-coenzyme A (CoA) and aceto-acetyl-CoA, generating 3-hydroxy-3-methylglutaryl (HMG)-CoA. The conversion of HMG-CoA to mevalonate by HMG-CoA reductase is the rate-limiting step in cholesterol biosynthesis. HMG-CoA reductase inhibitor medications, known as statins, inhibit the production of mevalonate, which is the precursor of cholesterol [137,138]. In addition, many studies have investigated their ability to inhibit the active form of oncoproteins, such as Rho and Ras pathways involved in the proliferation, migration, invasion, and survival of cancer cells [139,140].

The TCGA-KIRC project dataset revealed that HMG-CoA reductase gene expression is significantly lower in primary ccRCC compared to normal tissue [141]. Furthermore, a histologic examination of RCC primary specimens showed decreased levels of HMG-CoA reductase [142,143,144]. Therefore, cholesterol accumulation in ccRCC is likely the result of increased uptake rather than excessive biosynthesis from acetate.

To protect cells from the toxic effects of high free-cholesterol levels resulting from LAL hydrolysis, ACAT re-esterifies free cholesterol with FAs for storage inside the cell. High levels of CE in RCC tumors result from the increased activity of this enzyme [144].

In physiological conditions, as a consequence of insufficient intracellular lipid levels or following the stimulation of some growth factors, we observed the activation, via mammalian target of rapamycin (mTOR) signaling, of the sterol regulatory element-binding proteins (SREBPs) [142,144]. SREBPs are transcription factors that regulate enzymes involved in cholesterol and FA biosynthesis. Their activation requires proteolytic cleavage in the Golgi and translocation into the nucleus. Once inside the nucleus, they bind to the promoter regions of SREBP target genes and initiate the expression of enzymes involved in FA, TG, and cholesterol synthesis and uptake [145]. Additionally, it has been demonstrated that mTORC1 increases mitochondrial ATP generation and controls the translation of nucleus mitochondrial genes [146]. For instance, Liu et al. discovered that the LncRNA TP73-AS1 mediates the mTOR signal, encodes the nuclear mitochondrial protein SREBP1, produces ATP to enhance ccRCC cell proliferation, and suppresses apoptosis [32,144,145,146].

Recent research by Li et al. [147] provided an overview of the epigenetic control over SREBP signaling. They looked into the function of SETD8, a lysine methyltransferase (KMT) involved in the regulation of lysine residues in histones [148], which is implicated in a number of biological processes, including cell cycle progression [149], transcription activation [150], and DNA damage response [151].

According to their research, SETD8 significantly affected lipogenesis by methylating the 20th lysine of histone-4, which regulated the transcription of SREBP1 as a direct target. They discovered that SETD8 depletion stopped the development of kidney tumors in vivo, which was based on their observations of its impact on the growth of the mice xenograft model. Furthermore, RCC cells’ in vitro growth and metastasis were inhibited by the inhibitor UNC0379 and the siRNA downregulation of SETD8. Moreover, they demonstrated that USP17, an immediate early gene associated with a subfamily of cytokine-inducible deubiquitinating proteins (DUBs), was responsible for post-translational stabilization of the SETD8 protein by deubiquitination of the SETD8 protein [152]. Furthermore, by deubiquitinating Cdc25, which is connected to proliferation, as well as Snail and Twist, which are closely tied to metastasis, USP17, also known as DUB3, participates in the malignant transformation of cancer. Numerous studies have shown that USP17 is crucial for the development of many malignancies [147,153,154,155,156,157,158,159].

In 2022, Yang et al. [160] investigated the long-chain FA transferase CPT1A in ccRCC. CPT1A is decreased in ccRCC clinical samples and cell lines compared to normal samples. HIFs block CPT1A expression, reduce FA transport into mitochondria, and reroute FA to LDs for storage [161]. The authors utilized lentivirus overexpressing CPT1A to explore the neoplastic phenotypes of ccRCC, and the results showed that both tumor growth and lipid accumulation were suppressed in vitro and in vivo.

Due to enhanced cholesterol absorption and intracellular lipid accumulation caused by the expression of two class B scavenger receptors, CD36 and SRB1, in ccRCC cells, CPT1A deficiency promoted PI3K/Akt signaling activity and accelerated ccRCC cell growth. CD36 and SRB1 are members of the class B scavenger receptors [162]. CD36, counter-regulated by PPARα [163], is upregulated in ccRCC and promotes lipid uptake. SRB1 is a transmembrane protein well-characterized as a unique multifunctional receptor for cholesterol influx and efflux that mediates cholesterol movement into and out of cells [164].

PPARα is a crucial transcription factor that regulates the expression of proteins involved in FA uptake and β-oxidation [165]. PGC1α is a transcriptional coactivator within the metabolic reprogramming family [166]. PPARα and PGC1α stimulate the transcription of the CPT1A gene [167]. Yang et al. observed that CPT1A overexpression in cell lines enhanced the expression of PPARα and PGC1α.

In experiments with CPT1A-overexpressing cells, there were reduced levels of CD36, ABCG1, and SRB1 proteins and suppression of the activity of PI3K/Akt signaling with decreased intracellular lipid accumulation and cell apoptosis. Mechanistically, CPT1A overexpression downregulated CD36 and SRB1 and upregulated the expression of PPARα.

FAs, the primary components of lipids, have been found to function as substrates for energy storage, membrane formation, and signaling molecule creation. In the de novo synthesis of long-chain fatty acids (LCFAs), elongation and desaturation are the key stages. FA function and metabolic destiny are determined by the unsaturation degree and length. De novo lipogenesis yields palmitate as its primary product. It can be elongated and desaturated by SCD1 and ELOVLs to produce additional SFAs, MUFAs, and PUFAs such as oleate, palmitoleate, and stearate. These FAs, in turn, can be used to synthesize more complex lipids [49,120].

Zhang et al. [168] described how ccRCC cell lines under hypoxia upregulated SCD1 and that HIF-2α and SCD1 had synergistic effects in sustaining cancer cell survival and migration. These results were the consequence of a positive feedback loop between HIF-2 and SCD1, mediated by the activation of the PI3K/Akt pathway. Moreover, a recent study observed that SCD1 activity reduced cell viability and sensitized cancer cells to cisplatin-induced apoptotic death [49].

Tanaka et al. [169] investigated the involvement of elongases in RCC. Previous findings revealed an increased expression of ELOVL2 and ELOVL5 in ccRCC [115] and an *ELOVL2* role in promoting LD accumulation in cell lines [170]. They explored the correlation between ELOVL2 and ccRCC progression.

**Figure 3:**
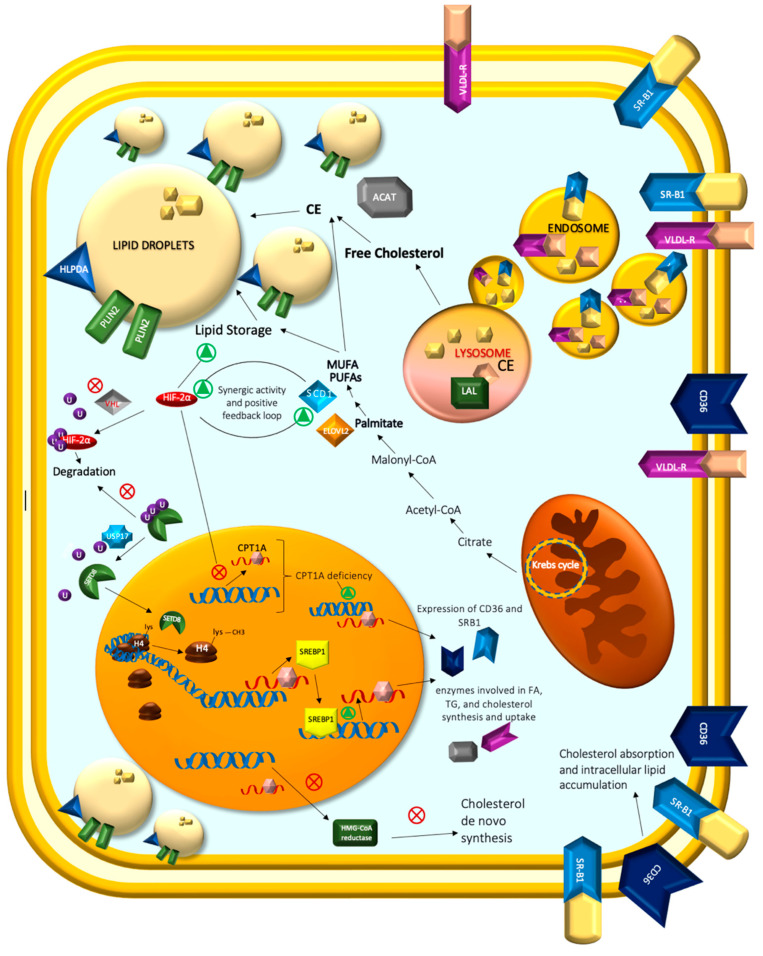
A hallmark of ccRCC is the accumulation of cholesterol, cholesterol esters, and other lipids collected in intracellular lipid droplets (LDs). LD-associated proteins, PLIN2 and HILPDA, are overexpressed in ccRCC, regulate lipid storage, and enrich lipids that contain polyunsaturated fatty-acyl side chains. Cholesterol arrives in RCC cells through direct uptake from the diet and can be acquired through receptors: very low-density lipoprotein receptor (VLDL-R) and scavenger receptor B1 (SR-B1). Once lipoprotein binds to its receptor, it forms an endosome within the membrane, which translocates into cells. Following internalization and transport into the lysosome, the cholesterol ester is hydrolyzed by lysosomal acid lipase (LAL) and upregulated in ccRCC to release the free cholesterol. To protect cells from the toxic effects of high free-cholesterol levels resulting from LAL hydrolysis, ACAT re-esterifies free cholesterol with FAs for storage inside the cell. High levels of CE in RCC tumors result from increased activity of this enzyme. Cholesterol de novo synthesis in ccRCC is reduced because of decreased levels of HMG-CoA reductase. Therefore, cholesterol accumulation in ccRCC is likely the result of increased uptake rather than excessive biosynthesis from acetate. SREBPs are transcription factors that regulate enzymes involved in cholesterol and fatty acid biosynthesis. Once inside the nucleus, they bind to the promoter regions of SREBP target genes and initiate the expression of enzymes involved in FA, TG, and cholesterol synthesis and uptake. SETD8 is stabilized by deubiquitination mediated by ubiquitin-specific protease 17 (USP17) and modulates the transcription of SREBP1 as a direct target by methylating the 20th lysine of histone-4 with enormous effects on lipogenesis. The long-chain fatty acid transferase CPT1A is decreased in ccRCC clinical samples and cell lines compared to normal samples. HIFs are responsible for inhibiting CPT1A expression, reducing FA transport into mitochondria, and rerouting FA to LDs for storage. CPT1A deficiency promotes the expression of two members of class B scavenger receptors, CD36 and SRB1, leading to increased cholesterol absorption and intracellular lipid accumulation. CD36 and SRB1 are members of class B scavenger receptors, which promote lipid and cholesterol uptake. Elongation and desaturation are the main steps of the de novo synthesis of long-chain FAs (LC-FAs). The length and degree of unsaturation are determinants of FA function and metabolic fate. Palmitate (16:0) is the main product of de novo lipogenesis. It can be elongated and desaturated through the activity of SCD1 and ELOVLs to generate additional SFAs, MUFAs, and PUFAs. ccRCC cell lines upregulate SCD1 and HIF-2α, and SCD1 had synergistic effects in sustaining cancer cell survival and migration. These effects were due to a positive feedback loop between HIF-2α and SCD1, mediated by PI3K/Akt pathway activation.

They showed that ELOVL2 ablation might suppress the production of docosahexaenoic acid (DHA) and LDs in renal cancer cells, suggesting that ELOVL2 overexpression may promote LD production through endogenous DHA production in RCC. Furthermore, ELOVL may promote endoplasmic reticulum (ER) stress and CHOP upregulation, downregulating BCL-2 and MCL-1. These findings suggested that ELOVL2 may protect cells against lipotoxicity-driven apoptosis by maintaining ER homeostasis to promote tumor growth and progression in RCC (Figure 3).

### 2.5. Arginine and miR-34a-5p/ASS1 Axis

The metabolism of cancer cells helps them adapt their pathological needs to fuel their accelerated proliferation and resistance to apoptosis. Arginine plays critical roles in many metabolic pathways, such as in the production of urea, nitric oxide (NO), and proline [171].

Ammonia from protein synthesis is transformed into urea by the hepatic urea cycle, which is a crucial detoxifying system [172]. The conventional cycle, on the other hand, functions as a “urea shunt” in the kidney. Large amounts of urea are normally excreted by the kidneys, which also create arginine that is exported to other organs. Tumors with altered expression of urea cycle enzymes, such as argininosuccinate synthase 1 (ASS1), argininosuccinate lyase (ASL), and arginase 2(ARG2), produce less nitrogen waste while diverting both carbon and nitrogen to the production of anabolic biomass [172,173,174,175,176].

Many malignancies have been shown to have a decrease in ASS1 expression, which favors cell proliferation by allowing the CAD (carbamoyl-phosphate synthase 2, aspartate transcarbamylase, and dihydroorotase) complex to activate pyrimidine synthesis [173,177].

Furthermore, NO is considered a potent regulator of numerous cellular processes, including growth and angiogenesis [178].

In a recent study, Khare et al. [175] investigated the loss of urea cycle enzymes ASS1 and ASL in ccRCC patients and elucidated the contributions of ASS1 and ASL loss toward ccRCC progression.

They reported a considerable growth inhibition in ccRCC cells with the simultaneous re-expression of ASS1 and ASL.

According to their findings, ccRCC tumors and cell lines had lower levels of NO metabolites and decreased NOS expression. They proposed that the depletion of ASS1 and ASL changes the cellular NO metabolism and promotes cell proliferation by controlling the cytotoxic effects of NO production.

In addition, they demonstrated that the expression of ASS1 and ASL modulates cellular nucleotide synthesis and aspartate levels. These findings show that ASS1 and ASL seem to be potential metabolic tumor suppressors in ccRCC, and that their loss conserves cellular aspartate pools and regulates NO production to offer ccRCC cells a proliferative advantage. ASS1- and ASL-deficient cells are arginine auxotrophs and must obtain their intracellular supply from external sources. Consequently, arginine deprivation has been considered as a promising potential treatment for malignancies lacking ASS1 and ASL [179,180].

ARG2 is persistently downregulated in ccRCC tumors relative to healthy tissue [181]. Reducing pyridoxal-5′-phosphate, an essential biosynthetic cofactor, and preventing a toxic buildup of polyamines [175,181] are two primary processes by which decreased ARG2 levels promote tumor growth. Consequently, the simultaneous downregulation of ASS1, ASL, and ARG2 contributes to the overall conservation of intracellular aspartate by preserving pyridoxal phosphate (PLP) and diverting aspartate away from the urea cycle.

Intriguingly, all three enzymes inhibit ccRCC cell growth in a catalytically dependent manner, as the re-expression of enzyme-dead mutants had no effect on the growth dynamics of ccRCC cells [175].

Further studies showed the role of competitive endogenous RNAs (ceRNAs) in the repression of ASS1 in ccRCC. Recent research by Poliseno et al. [182] demonstrates that pseudogenes can function as ceRNAs to modify other RNA transcripts in tumors by competing with miRNAs.

The microRNAs (miRNAs) are a class of noncoding RNAs that can bind to the 3′ untranslated region (3′UTR) of target gene transcripts to inhibit translation or reduce the stability of messenger RNA (mRNA) [183].

ASS1 is a target of miR-34a-5p and can be negatively regulated by miR-34a-5p [184].

Wang et al. [184] discovered that the androgen receptor (AR), which plays an essential role in tumor proliferation in cancer [185,186,187], could regulate ASS1 expression in RCC by regulating the ceRNA activity of ASS1P3, an ASS1 pseudogene.

In their findings, AR expression was negatively correlated with ASS1 expression, thus contributing to RCC tumor progression.

The pseudogene ASS1P3 could function as a ceRNA to regulate the expression level of its corresponding gene via miR-34a-5p. A decreased level of ASS1P3 expression led to the inhibition of ASS1 by miR-34a5p and increased cell proliferation.

Thus, the increased expression of ASS1P3 could reduce cell proliferation with a potential therapeutic modality for advanced RCC.

Although the authors did not detect a correlation between AR and ASS1P3 expression, they postulated that AR undergoes a physical interaction with ASS1P3 that inhibits ASS1P3’s interaction with miR-34a5p. Therefore, miR-34a5p might operate as an oncogene in this circumstance, and by reducing the expression of ASS1 and ASS1P3, it could partially reverse the AR-induced drop in ASS1 expression.

Similarly, Zeng et al. [188] recently described the involvement of lncRNA 00312 in the miR-34a-5p/ASS1 axis in RCC.

In their findings, lncRNA 00312 expression was significantly downregulated in RCC tissues. In addition, they showed that there were binding sites of miR-34a-5p in the 3′ region of lncRNA 00312.

Thus, lncRNA 00312, which is mostly localized in the cytoplasm of RCC cells, might function as a ceRNA to bind to microRNAs, thereby preventing microRNAs from inhibiting target gene transcripts.

The decreased expression of lncRNA 00312 in RCC was associated with a poorer prognosis according to tumor size, pathological grade, and TNM stage in RCC patients.

**Figure 4:**
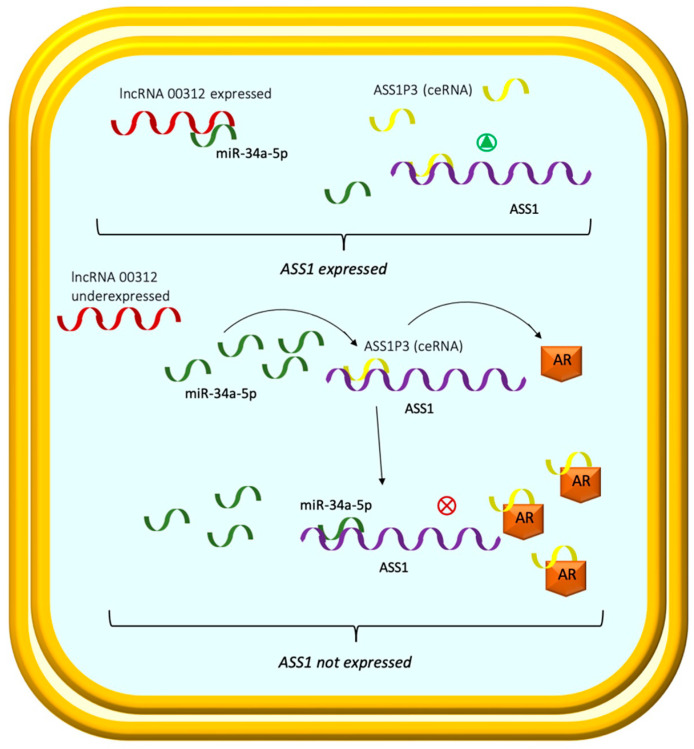
ASS1 can be negatively regulated by miR-34a-5p. The decrease in ASS1 expression favors cell proliferation. The pseudogene ASS1P3 could function as a ceRNA to regulate the expression level of its corresponding gene by competing with miR-34a-5p. A decreased level of ASS1P3 expression leads to the inhibition of ASS1 by miR-34a5p and increased cell proliferation. AR undergoes a physical interaction with ASS1P3 and regulates ASS1 expression in RCC by regulating the ceRNA activity of ASS1P3, contributing to RCC tumor progression. A decreased level of ASS1P3 expression leads to the inhibition of ASS1 by miR-34a5p and increased cell proliferation. lncRNA 00312 expression is significantly downregulated in RCC. Decreased expression of lncRNA 00312 in RCC is associated with a poorer prognosis.

By overexpressing lncRNA 00312 in RCC cells, the researchers discovered that lncRNA 00312, which induces an increase in ASS1, could reduce the proliferation, invasion, and death of RCC cells. These findings imply that lncRNA 00312 could be a viable therapeutic gene (Figure 4).

## 3. Metabolomic Biomarkers in Tumor Staging and Drug Efficacy

The in-depth understanding of RCC metabolome has provided new opportunities for biomarker detection. The main field of application has been the employment of these metabolites to stratify cancer subtypes and stages, and to evaluate drug sensitivity. Monitoring tumor progression through specific biofluid metabolite profiles has become a significant translational opportunity for urological cancers [189,190,191,192,193,194].

In a cohort of 61 patients with renal tumors and 68 healthy controls, Zhang et al. [195] used LC-MS to study the urine metabolomes.

Urine metabolic profiling could help distinguish RCC from benign renal tumors and healthy controls. With an AUC of 0.868 for the ten-fold cross-validation and 0.873 for the validation group, it was observed that a metabolite panel consisting of cortisol, testosterone, and L-2-aminoadipate adenylate had a high ability to discriminate RCC from benign tumors. With an AUC of 0.841 for the ten-fold cross-validation and 0.894 for the validation group, the panel of aminoadipic acid, 2-(formamido)-N1-(5-phospho-d-ribosyl) acetamidine, and alpha-N-phenylacetyl-l-glutamine was also able to identify the RCC group from the healthy control group.

According to a pilot study by Falegan et al. [196], urine and serum metabolomics may help distinguish between benign renal tumors and RCC, as well as for staging RCC. The pathological stage was used to stratify the malignant groups. In comparison to benign masses, they discovered RCC to have altered amounts of glycolytic and TCA cycle intermediates. For an ^1^H NMR-analyzed serum, OPLS-DA plots distinguished between benign masses versus pT1 (AUC = 0.83), benign masses versus pT3 (AUC = 0.87), and benign masses versus pT3 (AUC = 0.98) for urine samples. For the GCMS-analyzed serum, separation was seen between benign masses and pT3 (AUC = 0.93), pT1 and pT3 (AUC = 0.98), and benign masses and pT3 (AUC = 0.93) for urine samples.

Using urine-based metabolomics, Bifarin et al. [197] recently determined the RCC stage. A twenty-four-metabolite panel that successfully enabled RCC staging and distinguished between early RCC and advanced RC was obtained through a combined LC-MS and NMR method.

They discovered that when early RCC and advanced RCC groups were compared to healthy control samples, the levels of apo-[3-methylcrotonoyl-CoA:carbon-dioxide ligase (ADP-forming)], dihydrouridine, acetone, pyruvate, hydroxypropyl-asparagine, 7-aminomethyl-7-carbaguanine, and lys-gly/gly-lys increased. Additionally, these metabolites resulted in an increase when advanced RCC samples were compared to early RCC samples, suggesting that these metabolites could be used to distinguish between advanced and early RCC, as well as help distinguish both RCC stages and healthy controls.

Conversely, they discovered that early RCC was associated with lower levels of N, N-dimethyl-histidine, succinic anhydride, diethyl-2-methyl-3-oxosuccinate, cytosine dimer, 3-hydroxyanthranilic acid, and choline when compared to healthy controls. However, these levels grew in advanced RCC in comparison to control samples. Additionally, glycine, citrate, and 4-guanidinobutanoic acid had lower relative abundances in advanced RCC compared to early RCC. When early RCC samples were compared to healthy controls, both metabolites had larger relative abundances than when advanced RCC samples were matched with healthy controls (Table 2).

The acquisition of drug resistance is a crucial factor in RCC pharmacologic management and is responsible for the disease’s poor prognosis and limited long-term responses [198,199]. As a result, the first-line therapy for RCC includes tyrosine kinase inhibitors such as sunitinib, pazopanib, tivozanib, and cabozantinib, as well as mTOR inhibitors such as temsirolimus and everolimus [200,201]. Furthermore, thanks to the discoveries about the immunological background of RCC [202,203,204], a number of immune checkpoint inhibitors (ICIs) for the treatment of metastatic renal cell carcinoma (mRCC) were approved in recent years [205,206,207,208].

Recent studies [209,210,211,212,213] discovered the potential role of metabolic profiles in detecting biomarkers that predict drug efficacy to avoid these problems, reduce costs, and improve patient survival [214].

In 2018, Hatakeyama et al. [209] showed that intracellular levels of several metabolites involved in energy processes were found to be higher in sunitinib-resistant cell lines. Fructose 6-phosphate, D-sedoheptulose 7-phosphate, and glucose 1-phosphate levels were all significantly elevated in cells in the sunitinib-resistant condition. These findings suggest a faster rate of glycolysis and a larger absorption of glutamine into the TCA cycle. The increased expression of glutamine transporters in sunitinib-resistant cells supported this evidence. Additionally, the analysis suggests that resistant primary cells had significantly higher levels of glutathione and myo-inositol than sensitive cells, indicating that resistant cells had better antioxidant activity as a defense against sunitinib’s anticancer effects.

Jobard et al. [212] looked into the pretreatment and serial on-treatment sera of 121 patients enrolled in the French clinical trial TORAVA, in which 171 randomly selected patients with mRCC were treated with either a bevacizumab and temsirolimus combination or a standard regimen of either sunitinib or interferon-a plus bevacizumab [194]. Nuclear magnetic resonance spectroscopy was used to produce metabolic profiles, which were then compared during or between treatments.

Serum profiles were differentiated between before and after several weeks of treatment using a multivariate statistical analysis. After just two weeks of medication, the combination of bevacizumab and temsirolimus began to affect the patient’s metabolism more quickly than a normal treatment would. Lipids and carbohydrates were among the metabolites linked to the discrimination, which is consistent with the medicines’ known side effects and RCC metabolism [215]. The faster host metabolism modification shown in the experimental arm was caused by temsirolimus, according to a comparison of the metabolic profiles for the three arms. These outcomes demonstrated that medication responses and side effects might be predicted using a pharmacometabolomic technique.

The metabolic adjustments that underlie the success and failure of immunotherapy were studied by Mock et al. [211]. Based on the influence of the T-cell metabolism and a potential prognostic value demonstrated by transcriptomic analysis, they compared the serum lipid content between responders and nonresponders between the first and third cycles. They found that very-long-chain FA-containing lipids appeared to act as sensitizers to immune treatment. Similar to this, Li et al. [213] examined serum samples obtained from various RCC patients using LC-MS (at 4 and 8 weeks of treatment). An elevated kynurenine/tryptophan ratio was found via serum metabolite profiling. Kynurenine, a byproduct of tryptophan metabolism, suppresses the immune system. A lower OS rate for RCC patients was linked to the altered kynurenine/tryptophan ratio and an adaptative resistance mechanism.

In a recent study, Yao et al. [216] used 100 previously reported metabolic (MTB) pathways to quantify the metabolic landscape of the 729 ccRCC patients. Based on metabolism-related genes, they described three molecular subtypes of RCC and, using the principal component analysis (PCA), built the MTB score to calculate the metabolic pattern of individuals. Their results investigated the immune response and metabolic variations in the TCGA RCC cohort. They described how in individuals with ICI-resistance-associated pathways, the cluster with an unfavored prognosis demonstrated a high score of hypoxia and Wnt/beta-catenin signaling. These findings were consistent with previous studies which described HIF-related hypoxia and Wnt/beta-catenin signaling as pro-tumorigenic activities [217,218]. In this cluster, they described the highest immune and stromal scores, which may contribute to its poor outcomes. The cluster with better OS manifested a declined score of hypoxia and Wnt/beta-catenin signaling, which may be more likely to benefit from ICI therapy.

Variations in MTB scores were associated with cancer heterogeneity, treatment regimen, and clinical outcomes.

Patients who responded to ICI therapy and anti-VEGF medication had significantly higher MTB scores, demonstrating the prognostic value of this test. Overall, the data imply that patients with high MTB scores might benefit from ICI therapy and anti-VEGF medications [216], suggesting their potential role in therapy management.

## 4. Future Perspectives

The metabolomic approach used to study ccRCC biology has led to an increase in the knowledge of the pathological background of this disease. In addition, the integration with other omics branches has allowed for a more profound identification of a typical stage-related metabolic fingerprint.

In the last decade, metabolomics studies on the Warburg effect and lipidic metabolism described the main pathways potentially involved in the ccRCC signature.

Moreover, the discovery of lncRNAs implemented the knowledge of pathways. lncRNAs act as regulators, which can directly or indirectly exert a broad and complex influence on ccRCC metabolic pathways and products.

The integration of their role provided a basis for further research on tumor metabolism, the identification of new tumor markers, and potential therapeutic targets in the future.

In addition, a correct interpretation of the genetic landscape identified by these multiomics studies, even using artificial intelligence techniques, should help establish a better characterization of the pathways involved [219].

However, the results and experts’ interpretations of this complex network appear to be fragmentary due to the lack of a unique, standardized methodologic approach. Even though a significant amount of multiomics studies gave us new information about the stage-related ccRCC pathways, this knowledge needs to be corroborated by a sufficient correspondence between the findings on tissue samples and cell line samples [220,221,222,223]. To date, data presented in different studies present contradictions, and, despite evidence-based speculations describing metabolic networking, there is a lack of a comprehensive interpretation of studies.

A future perspective could be the standardization of methods used in this field of research in order to integrate and speculate deductions on standardized data through a unified approach.

Recently, the need to identify this systematization led to the publication of reviews and guides on reference materials, which are helpful as a baseline to calibrate, standardize, and compare the results obtained by different laboratories and represent an essential tool for future studies [223]. This is only the first step toward the endpoint of a unified protocol which could decrease the bias and heterogeneity risk in the methodological background.

The clinical application of this field is metabolic biomarker detection. This perspective improves disease management in diagnosis, follow-up, stage stratification, and therapeutic responsivity evaluation. However, more consistent studies on sensibility and specificity are needed to reach the point of everyday use of this biomarker.

Despite the application in different moments of disease management, the discovered impact of treatments on the metabolome of patients could lead to the use of biomarkers to assess the efficacy of the best treatment modality for metastatic stages.

Further studies are needed to validate the impact of the different treatments on the metabolic fingerprint in more extensive and independent clinical cohorts of different populations of ccRCC patients, stratified based on their ethnicity, age, sex, and staging, as well as complementing those studies with multiomics analyses to improve the accuracy in predictive biomarker detection and to give a better understanding of the mechanisms underlying cancer therapeutic resistance. In this regard, a better stratification of patients based on their metabolic fingerprints might help to predict the prognosis during the decision-making process of a pharmacological approach.

In this way, more consistent data could mean a promising clinical application in the next decade, embodying the new concept of personalized and targeted medicine.

## 5. Conclusions

In the last decade, an integrated approach using transcriptomics, metabolomics, and lipidomics has led to a better understanding of ccRCC as a metabolic disease. The comprehensive quantification of metabolic profiles in ccRCC patients would be an innovative strategy for choosing the optimal therapy for a specific patient. In addition, pharmacometabolomics represents a new field that could pave the way for novel stage-related and patient-tailored strategies according to the specific susceptibility to new classes of drugs. Further studies could provide a more consistent evidence-based application of this innovative perspective of disease management, improving the results in terms of OS and drug efficacy.

## Figures and Tables

**Table 1:** Summary of main altered metabolites and pathways in RCC.

Pathway	Metabolite	Sample	Variation in RCC	Reference
Glycolysis	Maltose	Tissue	↑	[46]
	Maltotriose	Tissue	↑	[46]
	Maltotetraose	Tissue	↑	[46]
	Maltopentaose	Tissue	↑	[46]
	Maltohexaose	Tissue	↑	[46]
	Fructose-6-phosphate	Tissue	↑	[46]
	Fructose-1,6-phosphate	Tissue	↑	[46]
	3-phosphoglycerate	Tissue	↓	[46]
	2-phosphoglyderate	Tissue	↓	[46]
	Phosphoenolpyruvate	Tissue	↓	[46]
	Lactate	Tissue	↑	[46,47,48]
		Urine	↑	[7]
	Pyruvate	Tissue	↑	[46,47,48]
		Urine	↑	[7]
TCA cycle	Malate	Tissue	↓	[47]
	Fumarate	Tissue	↓	[48]
	Citrate	Urine	↓	[7]
	L-2-hydroxyglutarate	Tissue	↑	[25]
Pentose Phosphate Pathway	Arabitol	Tissue	↓	[46]
	Xylitol	Tissue	↓	[46]
	Xylonate	Tissue	↓	[46]
	Glucose-6-phosphate	Tissue	↑	[46]
	Ribose-5-phosphate	Tissue	↑	[46]
	Ribulose-5-phosphate/xylulose 5-phosphate	Tissue	↑	[46]
	Sedoheptulose-7-phosphate	Tissue	↑	[46]
Biosynthesis of unsaturated FA	Arachidonate (20:4n6)	Tissue	↓	[49,50]
	Arachidate (20:0)	Tissue	↑	[49]
	Cis-vaccenate (18:1n7)	Tissue	↑	[49,50]
	Dihomo-linoleate (20:2n6)	Tissue	=/↑	[49,50]
	Dihomo-linolenate (20:3n3 or n6)	Tissue	↑	[49,50]
	Docosadienoate (22:2n6)	Tissue	↑	[49]
	Docosahexaenoate (22:6)	Tissue	↓	[49,50]
	Docosapentaenoate (22:5)	Tissue	=/↓	[49,50]
	Eicosapentaenoate (20:5n3)	Tissue	↓	[49,50]
	Eicosenoate (20:1n9 or 11)	Tissue	↑	[49]
	Erucate (22:1n9)	Tissue	↑	[49]
	Linoleate (18:2n6)	Tissue	↑	[49,50]
	Linolenate (18:3n3 or 6)	Tissue	↑	[49]
	Nervonate (24:1n9)	Tissue	↑	[49]
	Oleate (18:1n9)	Tissue	↑	[49]
	Palmitate (16:0)	Tissue	↑	[49,50]
	Palmitoleate (16:1n7)	Tissue	↑	[49,50]
	Stearate (18:0)	Tissue	↑	[49]
FA elongation	Palmitate (16:0)	Tissue	↑	[49]
Arachidonic acid metabolism	Arachidonate (20:4n6)	Tissue	↓	[44,49]
	5-HETE	Tissue	↓	[44,49]
	5-oxo-HETE	Tissue	↓	[44,49]
	PGE2	Tissue	↓	[44,49]
	Squalene	Tissue	↓	[44,49]
	7-alpha-hydroxycholesterol	Tissue	↓	[44,49]
	Desmosterol	Tissue	↓	[44,49]
Cholesterol biosynthesis	Cholesterol	Tissue	↓	[44,49]
	Cholesterol ester	Tissue	↑	[44,49]
	7-Dehydrocholesterol	Tissue	↓	[44,49]
	7-HOCA	Tissue	↓	[44,49]
	7-beta-hydroxycholesterol	Tissue	↓	[44,49]
	Tryptophan	Tissue Serum	↓	[45,50]
	Kynurenine	Tissue Serum	↓	[45,50]
	N-formylkynurenine	Urine	↑	[46,51]
Tryptophan metabolism	3-hydroxy-Lkynurenine	Urine	↑	[46,51]
	5-hydroxy-L-tryptophan	Urine	↑	[46,51]
	Serotonin	Urine	↑	[46,51]
	Acetyl-N-formyl-5-methoxy-kynurenamine	Urine	↓	[46,51]

Arrow indicates accumulation (↑) or deprivation (↓). (=) indicates no variation.

**Table 2:** Summary of potential biomarkers in RCC staging and drug efficacy evaluation.

	Metabolite	Sample	Variation	Ref.
Diagnosis/Staging			RCC vs. benign tumors	
	Cortisol	Urine	↑	[195]
	Testosterone	Urine	↑	
	L-2-aminoadipate adenylate	Urine	↑	
			RCC vs. healthy controls	
	Aminoadipic acid	Urine	↑	
	2-(formamido)-N1-(5-phospho-d-ribosyl) acetamidine	Urine	↑	
	Alpha-N-phenylacetyl-l-glutamine	Urine	↑	
			RCC vs. benign	[196]
	Citrate	Serum	↓	
		Urine	↓	
	Methanol	Serum	↓	
	Threonine Glycine	Serum	↓	
	Histidine Taurine	Serum	↓	
	Glutamine	Serum	↓	
	5-methylcitosine	Serum	↓	
	Eicosanoate	Serum	↓	
	Succinate	Urine	↓	
	Glycine	Urine	↓	
	3-hydroxybutyrate creatinine	Urine	↓	
	2-aminoisobutyrate	Urine	↓	
	Phenylalanine	Urine	↓	
	Methylhistidine	Urine	↓	
	Acetate threonine	Urine	↓	
	Gluconate	Urine	↓	
	Thymine	Urine	↓	
	Mannitol	Urine	↓	
	2-oxoisocaproate	Serum	↑	
	Creatine	Serum	↑	
	Isoleucine	Serum	↑	
	Glutamate	Serum	↑	
	Ornithine	Serum	↑	
	Tyrosine	Serum	↑	
	Octadecanoate	Serum	↑	
	Galactose	Serum	↑	
	Pyruvate	Serum	↑	
		Urine	↑	
	Lactate	Serum	↑	
		Urine	↑	
	Oxypurinol	Urine	↑	
	Gluconate	Urine	↑	
	Hypoxanthine	Urine	↑	
	Malonate	Urine	↑	
	Betaine	Urine	↑	
	Tryptophan	Urine	↑	
	Trigonelline	Urine	↑	
	Dimethylamine	Urine	↑	
	Glucose	Urine	↑	
	Erythritol	Urine	↑	
	2-oxoglutarate	Urine	↑	
	Myo-inositol	Urine	↑	
	Apo-[3-mthylcrotonoyl-CoA:carbon-dioxide ligase]	Urine	Early/Advanced RCC vs. healthy controls ↑	[197]
	Dihydrouridine	Urine	↑	
	Acetone	Urine	↑	
	Pyruvate	Urine	↑	
	Hydroxypropyl-asparagine	Urine	↑	
	7-aminomethyl-7-carbaguanine	Urine	↑	
	Lys-gly/gly-lys	Urine	↑	
	Apo-[3-mthylcrotonoyl-CoA:carbon-dioxide ligase]	Urine	↓ Advanced vs. early RCC ↑	
	Dihydrouridine	Urine	↑	
	Acetone			
	Pyruvate	Urine	↑	
	Hydroxypropyl-asparagine	Urine	↑	
	7-aminomethyl-7-carbaguanine	Urine	↑	
	Lys-gly/gly-lys	Urine	↑	
	N,N-dimethyl-histidine	Urine	Early RCC vs. healthy controls ↓	
	Succinic anhydride	Urine	↓	
	Diethyl-2-methyl-3-oxosuccinate	Urine	↓	
	Cytosine dimer	Urine	↓	
	3-hydroxyanthranilic acid	Urine	↓	
	N,N-dimethyl-histidine	Urine	Advanced RCC vs. healthy controls ↑	
	Succinic anhydride	Urine	↑	
	Diethyl-2-methyl-3-oxosuccinate	Urine	↑	
	Cytosine dimer	Urine	↑	
	3-hydroxyanthranilic acid	Urine	↑	
Drug Efficacy	Fructose-6-phosphate	Sunitinib Resistant Cell lines	↓	[209]
	D-sedoheptulose 7-phosphate		↓	
	Glucose 1-phosphate		↓	
	Lipid species	Serum	Bevacizumab+temsirolimus vs. sunitinib+interferon-alfa + bevacizumab ↑	
	Lipoproteins	Serum	↑	[212]
	Very-long-chain FA	Serum	Responders vs. nonresponders to immunotherapy ↑	[211]
	Kynurenine/tryptophan	Serum	↑	[213]

Arrow indicates accumulation (↑) or deprivation (↓).

## Data Availability

Not applicable.
